# The Effect of Gut Microbiota and Probiotics on Metabolism in Fish and Shrimp

**DOI:** 10.3390/ani12213016

**Published:** 2022-11-03

**Authors:** Einar Ringø, Ramasamy Harikrishnan, Mehdi Soltani, Koushik Ghosh

**Affiliations:** 1 Norwegian College of Fishery Science, Faculty of Bioscience, Fisheries and Economics, UiT The Arctic University of Norway, 9019 Tromsø, Norway; 2 Department of Zoology, Pachaiyappa’s College for Men, University of Madras, Kanchipuram 631 501, Tamil Nadu, India; 3 Department of Aquatic Animal Health, Faculty of Veterinary Medicine, University of Tehran, Tehran 1419963111, Iran; 4 Centre for Sustainable Aquatic Ecosystems, Harry Butler Institute, Murdoch University, Murdoch, WA 6150, Australia; 5 Aquaculture Laboratory, Department of Zoology, The University of Burdwan, Burdwan 713 104, West Bengal, India

**Keywords:** gut microbiota, probiotic administrations, lipid-, carbohydrate- and protein metabolism

## Abstract

**Simple Summary:**

The gastrointestinal microbiota and probiotic supplementations play a vital role in fish and shellfish health. Therefore, it is important to increase our knowledge and experience on their effect on lipid-, carbohydrate- and protein metabolism. Even though some information is available, further studies are needed to achieve sustainable aquaculture.

**Abstract:**

The present paper presents an overview of the effects of gut microbiota and probiotics on lipid-, carbohydrate-, protein- and amino acid metabolism in fish and shrimp. In probiotic fish studies, the zebrafish (*Danio rerio*) model is the most frequently used, and probiotic administration reveals the effect on glucose homeostasis, anti-lipidemic effects and increasing short-chain fatty acids, and increased expressions of genes related to carbohydrate metabolism and innate immunity, along with down-regulation of oxidative stress-related genes. Further, improved length of the intestinal villi and expression of nutrient transporters in fish owing to probiotics exposure have been documented. The present review will present an appraisal of the effect of intestinal microbiota and probiotic administration on the metabolism of nutrients and metabolites related to stress and immunity in diverse fish- and shrimp species. Furthermore, to give the reader satisfactory information on the topics discussed, some information from endothermic animals is also presented.

## 1. Introduction

Bacteria colonizing the gastrointestinal (GI) tract microbiota in fish and shellfish consists of allochthonous- and autochthonous bacteria [1]. The GI tract is colonized by numerous bacteria, which stimulate metabolic functions, GI development, improve digestion, enhance the immune response, and protect against exogenous bacteria and diseases [2,3,4], the development of metabolic syndrome [5], underpin host metabolic plasticity [6], and vitamin synthesis [7] and affect host health. The gut immune system involves three important defense mechanisms, (i) gut barriers, (ii) innate immunity, and (iii) acquired or adaptive immunity, which work together to improve disease resistance [8].

The intestinal bacterial community is modulated by internal and external factors, and these changes, both quantitative and qualitative, affect the health status [9,10,11,12]. It is generally accepted that aquatic animals do not have all the essential enzymes needed to handle dietary challenges. However, colonizing the GI tract by probiotic microorganisms secreting various digestive and degradation enzymes utilizing several nutritional compounds may enhance the utilization of undigestible carbohydrates as an energy source.

Several recent investigations have described that the intestinal microbiota reveals various biological effects and facilitates protein/amino acids (AAs) digestion and absorption by decomposing complex subunits, which are easy to absorb and consequently modify metabolic pathways on the host cell [13,14]. Studies have revealed that modulation of dietary protein/AAs is an approach for control of AAs utilization bacteria and their metabolic pathways, which may have an impact on host metabolism [15,16,17], and metabolites can modify the immune system and gene expression of host cells [18,19,20].

When discussing the GI tract microbiota of fish and their roles in metabolism and health, it is also of interest to notice that results from Atlantic salmon (*Salmo salar*) pyloric caeca indicate that *Carnobacterium*, a lactic acid bacterium, has an impact on flesh color [21]. Therefore, it is important to increase our knowledge and experience on gut bacteria colonizing and adhering to the GI tract of fish and shrimp to improve the growth performance and health of the host.

Regarding interactions between the gut microbiota and metabolism, the authors have included some information from endothermic animals.

## 2. Gut Microbiota and Lipid Metabolism

Two recent studies revealed that zebrafish (*Danio rerio*) intestine colonized by Firmicutes enhanced lipid bioavailability in the gut and liver [22,23]. Grass carp (*Ctenopharyngodon idella*) fed a ryegrass diet revealed that lipid metabolism was significantly improved by modulation in the gut microbiota [24], and the authors proposed the hypothesis that the metabolic role of the gut microbiota in carp was affected by dietary manipulation. Sheng et al. [25] revealed that zebrafish with *intact microbiota* improved the accumulation of lipids in the gut, and enhanced expression of *cox15*, *ppary*, and *slc2a1a*, genes related to lipid metabolism vs. that of germ-free fish. Hao et al. [26] revealed a rapid modulation of the hindgut microbiota of grass carp fed fish meal (FM) and Sudan grass (*Sorghum sudanense*) within 11 days. However, genes associated with lipid transport and metabolism were not significantly changed by the dietary shift. 

α-lipoic acid (α-LA) is an important antioxidant in the detoxification of oxygen species [27]. In a study using genetically improved farmed tilapia fed a high-saturated-fat diet (HFD), it was displayed that the adipose triglyceride gene was up-regulated in fish fed diet supplemented with 2.400 mg kg^−1^ α-LA, while diacylglycerol acyltransferase 2 gene was down-regulated [28]. In addition, a significant up-regulation of the fatty acid-binding protein gene in the liver by α-LA feeding was displayed. As modulation of the gut microbiota by α-LA feeding was not investigated in this study, one can speculate because, as in mice, α-LA and a high-fat diet modulated the gut microbiota [29].

In their study using yellowtail kingfish (*Seriola lalandi*), Ramirez and Romero [30] revealed a notable difference in the gut microbiota of wild and aquaculture-raised fish, as phylum *Firmicutes* was abundant in cultured fish in contrast to *Pseudomonadales,* which dominated in wild fish. Furthermore, lipid metabolism, biosynthesis of fatty acids, glycerolipid, glycerophospholipid, secondary bile acid, and sphingolipid were affected. Similar results on lipid metabolism were reported by Salas-Leiva et al. [31], analyzing the structure and metabolic contribution of gut microbiota in longfin yellowtail (*Seriola rivoliana*) juveniles.

Yildirimer and Brown [32] revealed significant enrichment of fatty acids and lipid metabolism genes in the allochthonous hindgut microbiota of Western Cascade rainbow trout compared to that of Eastern Cascade fish, even though a more diverse microbial community was revealed in Eastern Cascade fish.

Arias-Jayo et al. [33] revealed that in zebrafish fed the HFD, the dominant pathways involved were in AA metabolism in contrast to fish fed a high-saturated-fat DHA-enriched diet, where the pathways involved in lipid metabolism were prominent.

Meng et al. [34] revealed that waterborne copper exposure modulated the allochthonous microbiota and lipid metabolism in common carp (*Cyprinus carpio*) as intestinal *Roseburia* was positively associated with lipogenic enzymes, total protein, and triglycerides but negatively associated with lipolysis enzymes.

In a recent study with Nile tilapia (*Oreochromis niloticus*), Wu et al. [35] investigated the gill and GI tract microbiota and revealed a significant interdependence between specific gut bacteria and metabolites. For example, *Undibacterium*, *Crenothrix,* and *Cetobacterium*, potentially beneficial bacteria, were positively correlated with most intestinal metabolites, including lipid metabolism. In contrast, *Aeromonas*, *Acinetobacter*, Enterobacteriaceae, *Mycobacterium*, Clostridiales, and *Paeniclostridium* were negatively correlated to most intestinal metabolites. The dietary effect of essential oils on gilthead seabream significantly modulated the allochthonous gut microbiota, and the alterations in bacterial sequences affected lipid metabolisms [36].

It is generally accepted that the intestinal bacterial community is an important key regulating factor in lipid metabolism, and there is evidence available regarding the interaction between bile acids on lipid metabolism [37,38] and on the regulation of lipid metabolism by bile acid modification [39].

Grass carp fed a high dietary lipid diet supplemented with 0.06 g kg^−1^ bile acids modulated the intestinal microbiota by increasing the presence of *Cetobacterium*, which significantly increased the relative expression of lipid catabolism genes in muscle and hepatopancreas [37]. A recent study revealed that with supplementation of dietary bile acids, the transcription of lipogenic genes in tiger puffer (*Takifugu rubripes*) fed a *normal diet* decreased but increased the transcription of lipid digestion and lipid/cholesterol transport genes in fish fed control diet [40]. Dietary bile acids also significantly affect lipid metabolism by reducing lipid content in muscle and liver tissue, as well as the content of triglycerides and total cholesterol in the plasma of Nile tilapia [41]. Whether the increased transcription and the effect on lipid metabolism by bile acid supplementation revealed in these studies are related to changes in the gut bacterial community was not evaluated and merited further investigations.

G protein-coupled receptor (TGR5), involved in lipid metabolism, is activated by bile acids. For example, the administration of a TGR5 agonist reduced obesity in wild-type mice fed a high-lipid diet [42], but as it is unclear whether bile acids regulate lipid metabolism through TGR5 in fish, the topic merits investigation.

## 3. Gut Microbiota and Carbohydrate Metabolism

As the metabolism of carbohydrates is dependent on their source and the inclusion level of carbohydrates in the diet [43], it is important to know whether the gut microbiota can influence carbohydrate metabolism, related to the capacity of fish to utilize dietary carbohydrates as an essential supplement.

As FM and fish oil (FO) are important supplements, with increasing costs and shortages, the utilization of alternatives is becoming increasingly important, and plant feedstuffs are the most sustainable alternatives. When discussing the use of plant material, it is important to keep in mind that fish species consuming plant material (herbivores and omnivores) reveal greater diversity and abundance of intestinal bacteria than carnivores. The nutritive value of plant feedstuff is limited by a high content of non-starch polysaccharides (NSP), which cannot be utilized by fish, but low dietary inclusion levels of NSP may increase the abundance of bacteria displaying the ability to mobilize or inactivate NSP. For example, marine bacilli isolated from European sea bass (*Dicentrarchus labrax*) intestines were capable of hydrolyzing NSP [44], and 43 out of 160 isolates revealed high or broad carbohydrolytic capacity. Nine spore-forming isolates were efficient by metabolizing, i.e., xylose, galactose, arabinose, or mannose.

In humans, a clear context between gut microbiota and glucose metabolism disorders is revealed [45]. However, as this topic is less investigated in fish and shellfish, it merits investigation.

Wu et al. [35] revealed that intestinal microbes of Nile tilapia were positively correlated with most intestinal metabolites, including carbohydrate metabolism. In this study, *Cetobacterium*, a bacterial genus producing vitamin B_12_ and enhancing carbohydrate metabolism, was reported.

Juvenile rainbow trout fed either an experimental diet of hyperglucidic (40% gelatinized starch + 20% glucose) and hypoproteic (20%) diet or a high-protein (60%) glucose-free diet (control) revealed a rapid increase in gene expressions of glycolytic enzymes in juveniles fed the experimental diet relative to control diet, while genes involved in gluconeogenesis and AA catabolism were reduced [46]. Muscles from juveniles fed the hypoproteic diet displayed a down-regulation of glycolysis and glucose transport markers, as well as higher plasma glucose. Evaluation of the intestinal microbiota of yellowtail kingfish, wild and aquaculture, showed that the metabolism of carbohydrates, vitamins, and AA exhibited a difference in microbiota depending on the host origin [30]. A recent study revealed that the intestinal microbiota of longfin yellowtail juveniles, mainly dominated by Proteobacteria, Firmicutes, Bacteroides, Cyanobacteria, and Actinobacteria, exhibited a contribution to carbohydrate metabolism and AA metabolism [31].

## 4. Gut Microbiota and Protein and Amino Acid Metabolism

Dietary protein modulates AA utilization by gut microorganisms, and this has impacts on gut health [47]. For example, threonine is utilized by the gut for the synthesis of mucin and the maintenance of gut barrier integrity [48].

Gut microbiota either consume the AA synthesis from diet or AA building blocks of proteins in the host or metabolize nutrients via fermentation or conversion to form several metabolite compounds, including nitric oxide, ammonia, polyamines, hydrogen sulfide (H_2_S), indolic, and phenolic in both the proximal and distal intestine [47,49].

The GI tract microbiota can de novo synthesize some essential amino acids that contribute to the modification of AA homeostasis in the body [50]. At the same time, large amounts of AA and proteins undergo extensive metabolism by the intestinal bacteria or epithelial cells [50,51,52]. Digestive enzymes are formed, and nitrogen, immunoglobulins, urea, and mucins SI lumen, is further degraded, utilized, and metabolized by the intestinal microbiota.

The proteins are hydrolyzed into peptides and AAs by extracellular enzymes secreted by gut bacteria that enter the microbial cells [53]. AAs and peptide biomolecules can meet different fates [54]. For example, AAs catabolism is transamination or deamination formed by decarboxylases, and deaminases enzymes are involved in AAs metabolism to form biogenic/aromatic AAs/amines by decarboxylation through fission, deamination, decarboxylation, oxidation, and reduction or both (redox) to produce a variety of structurally related indoles and phenols [54].

### Protein/AA Metabolites in the Development of Gastrointestinal Diseases

The host response to the microbiota is essential in the homeostasis of intestinal immunity and inflammatory bowel disease (IBD), but defects in the intrinsic pathway did not affect homeostasis and the progress of IBD [55]. However, the interaction between the host and its intestinal microbiota is intricate. *Myxomycetes* and *Helicobacter* spp. are symbionts typically called pathogens that cause diseases under certain conditions [56]. It was a deliberated key factor of the host’s vulnerability to several diseases, including IBD [57]. Gut microbiota products and extreme protein fermentation formed various metabolites, including amines, hydrogen sulfide, p-cresol, and ammonia, which are impairment of colon epithelium (CE), induce intestinal leakage, DNA damage, IBD, colon cancer, etc. [58,59]. A high-protein diet (HPD) modifies the composition of the gut microbiota, augments substrate consumption rate, and increases protein volume content in the large, which maintains the metabolic homeostasis of CE [60]. Utilization of HPD and HFD modulated the intestinal microbiota and its biosynthesis metabolites, and this may lead to an imbalance result causing IIM that may further process IBD [61].

## 5. Probiotics and Metabolism

Increasing evidence during the last decade confirmed the crucial roles of probiotics (beneficial gut microbes) in host health [62]. Numerous metabolic functions could be under close regulation of probiotic organisms. Falcinelli et al. stated that “*probiotics can be used to positively alter gut microbiota, and their ability to improve metabolism*” [63]. The majority of the studies dealing with probiotics and metabolism have focused on either nutrient metabolism (lipid and glucose) [22,64] or metabolites linked with stress and immunity [65]. Nowadays, zebrafish have increasingly been used as a platform for developmental as well as biomedical research on disease modeling [66]. Consequently, the zebrafish model has gained acceptance for validating the beneficial functions of potential probiotics [67].

As evidenced, probiotics administration could be strongly allied to lowering cholesterol levels in vertebrate hosts [63,68]. In a previous report, Lye et al. [69] suggested five mechanisms by which probiotics may affect lipid metabolism: (1) assimilation of cholesterol during growth, (2) binding of cholesterol to cellular surface, (3) disruption of cholesterol micelle, (4) deconjugation of bile salt, and (5) bile salt hydrolysis activity. In a review devoted to probiotic products containing mainly live lactic acid bacteria (LAB), Cho and Kim revealed decreased total cholesterol and LDL cholesterol, but no significant effect on HDL cholesterol and triglycerides was reported [9]. In zebrafish larvae, administration of *Lactobacillus rhamnosus* IMC 501 downregulated the transcriptions of genes involved in cholesterol (*hnf4α* and *npc1l1*) and triglyceride (*fit2* and *mgll*) metabolism that decreased cholesterol- and triglyceride content along with increased fatty acid levels [11]. Further, adult zebrafish exposed to varying lipid levels revealed that high dietary lipid reduced the gut microbiota diversity, which affected the transcription of genes involved in appetite control, while supplementation of *Lb. rhamnosus* resulted in decreased total body cholesterol [70]. In adult zebrafish, administration of *Lb. rhamnosus* alleviated per-fluoro-butane-sulfonate (PFBS) induced gut microbial dysbiosis and lipid metabolism disorders [71]. In a follow-up study, the authors noted antagonistic interaction between PFBS and *Lb. rhamnosus* on glucose metabolism. PFBS alone induced elevated blood glucose levels in male zebrafish; however, probiotic supplementation led to increased insulin levels that reduced glucose accumulation and enhanced ATP production [62]. In addition, oral administration of *Lb. rhamnosus* on Type 2 diabetes mellitus (T2DM) was investigated in T2DM-induced adult male zebrafish. The results revealed that probiotic administration decreased the blood glucose level by decreasing pro-inflammatory cytokines responsible for signaling in T2DM [72].

In zebrafish, administration of *Chromobacterium aquaticum* for 8 weeks enhanced growth and metabolism, as a significant increase in expression of glucokinase, hexokinase, glucose-6-phosphatase, and pyruvate kinase and growth hormone receptor and insulin-like growth factor-1 were noticed [67]. Furthermore, *C. aquaticum* supplementation modulated innate immunity-related genes (IL-1β, IL-6, TNF-α, IL-10, IL-21, NF-κb, lysozyme, and complement C3b) and improved survivability in zebrafish challenged against *Aeromonas hydrophila* and *Streptococcus iniae*. Feeding xylanase-expressing *Bacillus amyloliquefaciens* R8 to zebrafish improved hepatic glucose, lipid metabolism, and reduced oxidative stress and immunity, and enhanced resistance against *A. hydrophila* and *S. agalactiae* [65]. In this study, increased expressions of glycolysis-related genes (e.g., hexokinase, glucokinase, glucose-6-phosphatase, and pyruvate kinase) and elevated levels of 3-hydroxyacyl-CoA dehydrogenase and citrate synthase (associated with β-oxidation and mitochondrial integrity) activities were also recorded. Further, decreased expressions of oxidative stress-related genes (SOD, Gpx, NOS2, and Hsp70) and an apoptotic gene (tp53), along with increased expression of an anti-apoptotic gene (bcl-2) and innate immunity-related genes (IL-1β, IL-6, IL-21, TNF-α, and TLR-1, -3, -4) were also noticed in probiotics-fed zebrafish suggesting the probiotic potential of *B. amyloliquefaciens* R8 [65]. Zebrafish-fed diets supplemented with transgenic phytase-expressing probiotic *Bacillus subtilis* revealed significant improvement in gene expression for appetite, peptide transport, somatic growth, and bone metabolism [73]. The likely mechanisms through which dietary supplementation of probiotics can exert favorable effects on glucose homeostasis and anti-lipidemic impacts could be related to increasing the short-chain fatty acid (SCFA) levels and metabolites with antimicrobial, anti-inflammatory, and immunomodulatory properties (e.g., bacteriocins, vitamins K and B_2_) [63,68,74]. Probiotics could also be linked to improved growth by increasing lipid catabolism through β-oxidation that promotes intestinal absorption of fatty acids metabolites [75]. Apart from zebrafish, probiotic efficiency linked with metabolic function has also been illustrated with other teleosts and shrimps. Olive flounder (*Paralichthys olivaceus*) fed *Bacillus clausii* displayed higher growth and feed efficiency vs. fish-fed control diets [76]. Oral administration of *Shewanella putrefaciens* (viable/non-viable) revealed improved growth, metabolism, immune response, and disease resistance in gilthead seabream (*Sparus aurata*) and Senegalese sole (*Solea senegalensis*) [77]. Lyophilized cells of *S. putrefaciens* significantly increased linolenic acid (C18:3 n-3) and linoleic acid (18:2 n-6) in the liver in juvenile Senegalese sole [78]. Short-term (30 d) exposure to *Enterococcus faecalis* FC11682 also increased both linolenic acid and linoleic acid levels in Malaysian mahseer (*Tor *tambroides**) post larvae [79]. Linolenic acid serves as a precursor for eicosapentaenoic acid (20:5 n-3) and docosahexaenoic acid (22:6 n-3), while linoleic acid for arachidonic acid (20:4 n-6) as a precursor for eicosanoids, which are integral for cellular and metabolic activities including membrane integrity, gene regulation and immune response [79,80]. A study using Senegalese sole larvae demonstrated that *S. putrefaciens* administration induced changes in the expression of carboxypeptidase A1, trypsinogen, cathepsin Z, and proteasome 26S non-ATPase subunit 3 involved in digestion and metabolic functions [81].

Effects on immunity, feed intake, appetite, and nutrient absorption owing to dietary supplementation of probiotics might be correlated with the overall metabolic function of the host. Dietary *Lb. acidophilus* resulted in the up-regulation of TNF-1α and TNF-2α and the down-regulation of appetite-related gene expression in goldfish (*Carassius auratus gibelio*) [82]. In gnotobiotic European sea bass (*Dicentrarchus labrax*) larvae, *Vibrio lentus* induced expression of genes related to cell proliferation, cell death, iron transport, and cell adhesion [83]. In addition, the up-regulation of immune genes in larvae exposed to *V. lentus* has also been recorded. *B. subtilis* supplementation improved growth, anti-inflammatory, and antioxidant properties of juvenile Yoshitomi tilapia challenged against pathogenic *A. hydrophila* [84]. Furthermore, serum lysozyme, alkaline phosphatase, superoxide dismutase, and catalase activities were improved, while serum aspartate-aminotransferase, alanine-aminotransferase, malondialdehyde, and C3 complement were reduced due to administration. It is reported that *B. subtilis* E20 improved the immune response in white shrimp (*Litopenaeus vannamei*) through glutamine metabolism and the hexosamine biosynthesis pathway [85]. Shrimp fed *B. subtilis* E20 increased absorption of AAs, including glutamine, and the authors suggested that *B. subtilis* E20 promotes the digestibility of glutamine, and the increased glutamine content in shrimp can be used as fuel for immune cells. Administration of *Lactococcus lactis* Z-2 enhanced metabolism in common carp (*Cyprinus carpio*) through the expression of Sglt1, Glut2, Pept1, rBAT along with modulation of immunity, antioxidant status, and disease resistance [86]. Application of *B. tequilensis*, diets or water, enhanced growth, nutrient utilization, and non-specific immunity in rohu (*Labeo rohita*) [87]. Administration of *B. safensis* NPUST1 in a Nile tilapia diet for 8 weeks resulted in increased hepatic mRNA expressions for glucose metabolism and growth-related genes (GK, G6Pase, GHR, and IGF-1) and significantly induced immune parameters in head kidney leukocytes (e.g., phagocytic activity, respiratory burst, and superoxide dismutase activity), serum lysozyme, and expression of immune- genes in the head kidney and spleen [88]. Improved length of the intestinal villi in probiotics-exposed fish might be associated with increased nutrient absorption contributing to an improvement in feed utilization and metabolic efficiency. Administration of *Lb. rhamnosus* to Nile tilapia significantly improved growth and the length of intestinal microvilli [89]. More recently, Salam et al. [90] demonstrated that dietary supplementation of probiotics (*Enterococcus xiangfangensis*, *Pseudomonas stutzeri*, *B. subtilis*, and a consortium of five gut bacteria) in silver barb (*Barbonymus gonionotus*) promoted growth, improved the length of intestinal villi, and reduced the number of pathogenic bacteria in the intestine. In Nile tilapia, supplementation of *B. amyloliquefaciens* to a high-carbohydrate diet increased acetate-producing bacteria, reduced mesenteric fat index, and lowered lipid deposition in the liver by increasing SCFAs in the intestinal content [91].

It has been indicated that probiotics influence metabolome in fish. Dietary administration of host-derived probiotics *Lc. lactis* WFLU12 revealed that 53 out of 200 metabolites from intestinal luminal metabolome and 5 out of 171 metabolites from serum metabolome, respectively, were present in significantly higher concentrations in probiotic-fed olive flounder than in the control group [75]. Several metabolites, e.g., citrulline, intermediated of the tricarboxylic acid cycle, SCFAs, vitamins, and taurine, were significantly higher in the probiotic-fed group than in the control group. Although underlying mechanisms behind the influence have not been clearly understood in most studies, available evidence presented in Table 1 suggests the influence of the probiotics on diverse metabolic processes in fish.

*Cetobacterium*, a Gram-negative, rod-shaped, non-spore-forming and non-motile bacteria, are revealed in the GI tract of several fish species, e.g., giant Amazonian fish (*Arapaima gigas*) and grass carp [92,93], and two recent studies have displayed that supplementation of a fermentation product of *Cetobacterium somerae* XMX-1 to common carp affected genes related to lipid metabolism-related genes [94,95].

## 6. Conclusions and Further Directions

Since Ganguly and Prasad [96] published their review entitled: *Microflora in fish digestive tract plays a significant role in digestion and metabolism*, knowledge about the gut microbiota and metabolism, and regulation of genes involved has increased in fish and shrimp during the last years, but several issues merit further investigation. For example, a controversial hypothesis can be put forward as the gut microbiota affect the flesh color [21]. Can the gut microbiota affecting flesh color also affect metabolism? This topic merits investigation. Furthermore, bile acid is important in lipid metabolism; however, as less information is available on the dietary effect of bile acid on the intestinal microbiota and lipid metabolism [37], the topic merits investigation.

Studies have revealed that the administration of *C. somerae* affected lipid metabolism-related genes in common carp [94,95], but what about its effect on carbohydrate-, protein-, and AA metabolism? This merits investigation.

In a review entitled “*Probiotic mechanisms affecting glucose homeostasis*,” Pintaric and Lagerhole [97] recommended that the effect of probiotics on glucose and its homeostasis merits further investigations, a topic which should be of high interest to aquaculture scientists.

Endothermic investigations have shown that docosahexaenoic acid and eicosapentaenoic acid can reverse the imbalance of the intestinal bacterial community by enhancing *Bifidobacterium*, *Lactobacillus*, and butyrate-producing bacteria, *Roseburia* and *Coprococcus*, a topic that merits investigations in fish and shellfish. As the GI tract of fish reveals great diversity [98], we must clarify if this diversity can impact the interactions between the gut microbiota and metabolism.

Nutrition regulates gut microbiota composition and function by influencing microbial diversity, immune functions, macromolecule metabolism, energy harvest, intestinal barrier permeability, and enzyme activities. Evidence from existing studies emphasizes the importance of adequate and balanced nutrition regarding energy and macronutrient components for gut microbiota.

## Figures and Tables

**Table 1:** Effects of probiotics on the metabolism of finfish.

Finfish Species	Bacteria Species	Effects	References
Senegalese sole (*Solea senegalensis)* (J)	Lyophilized cells of *Shewanella putrefaciens*	↑linolenic acid (18:3 n-3) and linoleic acid (18:2 n-6) in liver	[78]
Japanese flounder (*Paralichthys olivaceus)*	*Bacillus clausii* + fructo- and/ or mannan oligosaccharide	↓body lipid deposition, triglyceride, low-density lipoprotein, cholesterol	[76]
Nile tilapia, (*Oreochromis niloticus)*	*Lactobacillus rhamnosus*	↑growth, length of intestinal microvilli	[89]
Zebrafish (*Danio rerio*)	Intestinal microbiota	↑Uptake of fatty acid and lipid droplet formation in the gut epithelium and liver	[22]
Zebrafish larvae	*Lb. rhamnosus*	↑short-chain fatty acids; ↓triglycerides and cholesterol; ↑length of microvilli, height of enterocytes	[11]
Goldfish (*Carassius auratus gibelio)*	*Lactobacillus acidophilus*	↑immune-genes expression (TNF-1α and TNF-2α); ↓appetite related gene expression	[82]
Zebrafish (A)	*Lb. rhamnosus* + different lipid levels	↓transcription of genes in cholesterol- and triglyceride metabolism	[70]
Gnotobiotic European sea bass (*Dicentrarchus labrax)* larvae	*Vibrio lentus*	↑expression of genes for cell proliferation, cell death, metabolism, iron transport, cell adhesion, and immune genes	[83]
*Yoshitomi tilapia* (J)	*Bacillus subtilis*	↑anti-inflammatory and antioxidant properties, serum lysozyme, alkaline phosphatase, superoxide dismutase, and catalase activities ↓serum aspartate aminotransferase, alanine aminotransferase, malondialdehyde, and C3 complement	[84]
Olive flounder (*Paralichthys olivaceus)*	*Lactococcus lactis* WFLU12	↑citrulline, tricarboxylic acid cycle intermediates, SCFAs, vitamins, and taurine	[75]
Senegalese sole larvae	*S. putrefaciens*	↑expression of genes (carboxypeptidase A1, trypsinogen, cathepsin Z, and proteasome 26S non-ATPase subunit 3)	[81]
Zebrafish	*Bacillus amyloliquefaciens* R8	↑expressions of glycolytic genes (hexokinase, glucokinase, glucose-6-phosphatase, and pyruvate kinase), enzyme activities for fatty acid β-oxidation (3-hydroxyacyl-coenzyme A dehydrogenase and citrate synthase)	[65]
Zebrafish	*Chromobacterium aquaticum*	↑mRNA expression glucokinase, hexokinase, glucose-6-phosphatase, and pyruvate kinase	[67]
Zebrafish	Transgenic phytase-expressing probiotic, *Bacillus subtilis*	↑expression of genes for appetite, peptide transport, somatic growth, and bone metabolism (bglap)	[73]
Gilthead seabream (*Sparus aurata)* and Senegalese sole	*Shewanella putrefaciens* (known as Pdp11 or more recently as SpPdp11)	↑carboxypeptidase A1 (cpa1), trypsinogen (tryp1), cathepsin Z (ctsz) and proteasome 26S non-ATPase subunit3 (pmsd3)	[77]
Zebrafish (A)	*Lb. rhamnosus +* perfluorobutanesulfonate (PFBS)	↑Fatty acid synthesis and β-oxidation (♀) and accumulation of triglyceride in the liver (♂)	[71]
Zebrafish (A)	PFBS and probiotic bacteria, *Lb. rhamnosus*	↑hepatic hypertrophy, blood glucose, ATP production (♂), and insulin level	[62]
Common carp (*Cyprinus carpio)*	*Lc. lactis* Z-2	↑expression of nutrient transporters (Sglt1, Glut2, Pept1, rBAT), immunity and antioxidant status	[86]
Rohu (*Labeo rohita)* fingerlings	*Bacillus tequilensis*	↑growth, nutrient utilization, and non-specific immunity	[87]
Nile tilapia	*Bacillus safensis* NPUST1	↑hepatic mRNA expressions for glucose metabolism and growth-related genes (viz., GK, G6Pase, GHR, and IGF-1), phagocytosis, respiratory burst, superoxide dismutase activity (head kidney), serum lysozyme, and expression of immune- genes (head kidney and spleen)	[88]
Silver barb (*Barbonymus gonionotus)*	*Enterococcus xiangfangensis*, *Pseudomonas stutzeri*, *B. subtilis* and a consortium of five gut bacteria	↑ growth, length of intestinal villi	[90]
Zebrafish (A) Type 2 diabetes mellitus induced (♂)	*Lb. rhamnosus*	↓ blood glucose, and pro-inflammatory cytokines	[72]
Malaysian mahseer *(Tor tambroides)*	*Enterococcus faecalis* FC11682	↑linolenic acid and linoleic acid	[79]
Nile tilapia	*B. amyloliquefaciens*	↓mesenteric fat index, lipid deposition (liver), ↑SCFAs (intestine)	[91]

F—fry; J—juvenile; A—adult; ↑—positive effect; ↓—negative effect.

## Data Availability

Not applicable.
